# Modulation of visual attention by object affordance

**DOI:** 10.3389/fpsyg.2014.00059

**Published:** 2014-02-06

**Authors:** Patricia Garrido-Vásquez, Anna Schubö

**Affiliations:** Experimental and Biological Psychology, Philipps University MarburgMarburg, Germany

**Keywords:** object affordance, attention, motor, graspability, reachability

## Abstract

Some objects in our environment are strongly tied to motor actions, a phenomenon called object affordance. A cup, for example, affords us to reach out to it and grasp it by its handle. Studies indicate that merely viewing an affording object triggers motor activations in the brain. The present study investigated whether object affordance would also result in an attention bias, that is, whether observers would rather attend to graspable objects within reach compared to non-graspable but reachable objects or to graspable objects out of reach. To this end, we conducted a combined reaction time and motion tracking study with a table in a virtual three-dimensional space. Two objects were positioned on the table, one near, the other one far from the observer. In each trial, two graspable objects, two non-graspable objects, or a combination of both was presented. Participants were instructed to detect a probe appearing on one of the objects as quickly as possible. Detection times served as indirect measure of attention allocation. The motor association with the graspable object was additionally enhanced by having participants grasp a real object in some of the trials. We hypothesized that visual attention would be preferentially allocated to the near graspable object, which should be reflected in reduced reaction times in this condition. Our results confirm this assumption: probe detection was fastest at the graspable object at the near position compared to the far position or to a non-graspable object. A follow-up experiment revealed that in addition to object affordance *per se*, immediate graspability of an affording object may also influence this near-space advantage. Our results suggest that visuospatial attention is preferentially allocated to affording objects which are immediately graspable, and thus establish a strong link between an object’ s motor affordance and visual attention.

## INTRODUCTION

Gibson (1979) coined the concept of object affordance in his ecological approach to perception. In a nutshell, affordances are what the environment offers to the observer, or in other words, what we can do with the objects surrounding us. For instance, a solid object of a certain size and/or furnished with a handle may afford us to grasp it, and this potential of the object for action is already present in the visual array. The concept of object affordance therefore establishes a strong connection between visual perception and motor behavior.

In recent years, neuroscientific research has been dedicated to unraveling the neural mechanisms of object affordance. One seminal study (Chao and Martin, 2000) compared the processing of different types of stimuli (faces, houses, animals, and tools) in a functional magnetic resonance imaging (fMRI) experiment. Tools, which can be considered a stimulus category that affords grasping, elicited stronger activations in the left premotor and posterior parietal cortices than other stimulus types. These regions are associated with moving one’s hand and with grasping, respectively. Remarkably, these activations occurred in the absence of any task, and were observed even though the tools were only presented in the form of photos on a screen. These results therefore indicate that the implication of the tools for action was processed independently of any intention or possibility to interact with them. More recently, Proverbio et al. (2011) confirmed these results in an event-related potentials (ERPs) study, in which passive viewing of tool pictures elicited an enhanced left-frontal negativity compared to non-tools, starting about 210 ms after picture onset. This activity was localized in motor regions, namely bilateral premotor cortex and left post-central gyrus using an ERP source reconstruction method. These lines of research, together with results from related studies (Creem-Regehr and Lee, 2005; Handy et al., 2006) provide evidence of a neural link between visual processing on the one hand and motor-related activations on the other in object affordance. This link has also been referred to as visuomotor response (Handy et al., 2003; Gallivan et al., 2011).

Handy et al. (2003) put forward the idea that one possible consequence of this previously described visuomotor response may be that more attention is allocated to affording objects. In their study, the authors presented line drawings of two objects (one graspable and one not) simultaneously, either left and right or above and below from fixation. This design was referred to as object competition model by the authors, because it pits two objects against each other. A target was subsequently superimposed on one of the two objects, and participants were instructed to detect these targets as quickly as possible. ERP data indicated a sensory gain for graspable objects, with increased P1 amplitudes for targets superimposed on tools compared to non-tools, but only when the targets appeared on tools in the right or in the lower visual field. Target detection latencies in the reaction times were significantly decreased for tools in the lower hemifield. The authors therefore concluded that tools automatically attract attention when they appear at locations important for grasping (Handy et al., 2003). However, in latter study the temporal delay between the onset of the object pictures and the targets to which reaction times and ERPs were measured was at least 650 ms long. Evidence suggests that selective visual attention is allocated much faster, namely within the first 200 ms of processing (Wykowska and Schubö, 2012). Therefore, the possibility arises that in the study by Handy et al. (2003) other processes than the initial allocation of selective visual attention were captured. For example, the attentional interpretation of the data is at odds with the absence of a significant reaction time advantage for target detection at the tool on the right, both in comparison to a non-tool at the same position, and with the tool-left condition. Therefore, further investigation using shorter delays between stimulus and target presentation is warranted.

In line with the findings by Handy et al. (2003), who reported that affording objects may attract attention as a function of their spatial location, some studies suggest that distance of an object from the observer could be a mediating factor in object affordance. More precisely, a tool may be more affording when it is close to the observer (peripersonal space) rather than located farther away and not immediately reachable (extrapersonal space). For example, Gallivan et al. (2009, 2011) reported that a region in superior parieto-occipital cortex (SPOC) reacts more strongly to objects immediately reachable with the right hand (for right-handers) compared to more distant objects or objects within immediate reach of the left hand but not the right, even during passive viewing. The authors suggest that SPOC encodes the potential of immediately acting on objects, which relates to their affordance (Gallivan et al., 2009). The role of object distance has also been corroborated in a TMS study by Cardellicchio et al. (2011), who reported significantly enhanced motor evoked potentials (MEPs) at participant’s right hands when a graspable object (a cup) was presented in near space, compared to the presentation of a non-graspable object (a large cube) at the same location. However, in far space, this MEP difference was no longer significant, while the MEP elicited by the cup in near space was significantly greater than the MEP elicited by the cup in far space. In a similar vein, another study found significant congruency effects for pantomime movements in near space, depending on whether the handle orientation of a cup was congruent with the hand the participant was supposed to move. There were, however, no such congruency effects in far space (Costantini et al., 2010, 2011a). Furthermore, evidence shows that at the level of semantics, possible interactions with an object are triggered faster when these objects are immediately reachable than when not (Costantini et al., 2011b). Thus, experimental findings suggest that object affordance and the visuomotor response triggered by an object may be modulated by its distance to the observer. What has not been investigated so far is whether attention deployment to a graspable object is also modulated by object distance.

### RATIONALE OF THE STUDY

**FIGURE 1:**
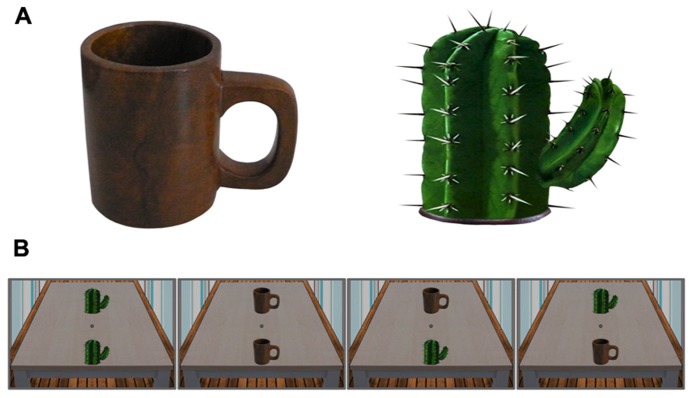
**Objects used in the present experiment.** **(A)** Cup (left) and cactus (right), shown here without background. **(B)** Stimuli in the probe detection task. Two objects appeared on the table on screen, one of which changed its luminance 200 ms after stimulus onset. The two left displays represent the identical-objects conditions, the two right displays show the different-objects conditions.

We investigated attentional deployment to affording and non-affording objects as a function of their graspability and reachability. To this end, we applied a task similar to the one used by Handy et al. (2003) with two objects presented simultaneously and a probe subsequently appearing on one of them. In order to avoid physical imbalances induced by lateralized stimulus presentation, objects were presented on the vertical meridian. To create an impression of depth, a virtual three-dimensional space was designed, depicting a table in a room. Due to their arrangement at the front versus at the back of the table one of the objects appeared close, the other one far from the observer. We used luminance change of the whole object as probe in order to avoid emphasizing a particular part of the object. A cup was used as an affording object and a cactus represented the non-affording category (see **Figure 1**).

In line with the procedure of previous studies (Gallivan et al., 2009, 2011) and in order to strengthen the motor association with the cup, grasp trials in which participants interacted with a real cup were included in the experiment. The cup on which participants performed the grasp trials was identical to the one presented on screen, such that haptic experience could be transferred to the cup on screen. Recent research has outlined the importance of haptic experience with objects, specifically when visual information is incomplete (Takahashi and Watt, 2012). This was also the case in our experiment, because even though the stimuli presented on screen were designed to appear as realistic as possible, they cannot achieve the level of visual completeness of a real cup. Furthermore, responding to graspable objects with button presses is a rather arbitrary action and may interfere with lifelong experience of interacting with objects (Handy et al., 2003; Valyear et al., 2012). Thus, interspersing the probe detection task with trials in which a natural interaction with the object is performed constantly reminds participants of the cup’s real function.

Probe detection has been repeatedly used as a means to measure the allocation of visuospatial attention. The idea behind is that probe detection is speeded up when the probe appears at a currently attended location (Posner, 1980; Humphreys et al., 2004; Wykowska and Schubö, 2010). As discussed above, the delay between stimulus presentation and probe appearance in Handy et al. (2003) may be too long to tap initial allocation of visual attention. Therefore, it is unclear what the reported reaction time advantage for tools presented in the lower or right visual hemifield may actually have reflected. Building upon previous evidence (Wykowska and Schubö, 2012), we assumed that a 200 ms delay between stimulus and probe onset would be suitable to test initial allocation of attention in the present experiment. If affording objects in peripersonal space selectively attract initial attention, we would expect reaction times for probes appearing at the cup in near space to be faster than for the cactus at the same position. Furthermore, probe detection at the cup in near space should be faster than at the same object in far space. These predictions were tested and confirmed in Experiment 1. In a second experiment, we additionally varied immediate graspability by means of handle orientation. Based on studies relating an object’s grasp affordance to its immediate reach- and graspability with the right hand in right-handers (Buccino et al., 2009; Gallivan et al., 2009, 2011), the near cup with its handle to the left should not attract more attention than the cactus at the same position, and there should be no significant difference between probe detection at the near and far cup position with handles facing to the left.

## EXPERIMENT 1

In Experiment 1 we sought to determine whether the affording object (a cup) located in near space attracts more visuospatial attention than (1) the same object presented in far space, and (2) the non-affording object (a cactus) in near space. This was measured indirectly with a probe detection task, interspersed with grasp trials.

### MATERIALS AND METHODS

#### Participants

Thirty-nine students from the Philipps University Marburg participated in the experiment for course credit. Data sets from four participants were discarded, one due to technical problems during the measurement, one because of too many errors (11% of the trials, which is four standard deviations above the group mean), and two because of very slow responses (reaction time more than two standard deviations above the overall reaction time mean of all participants). The remaining 35 participants (23 female) had a mean age of 22.91 years (*SD* = 2.70) and were right-handed according to the Edinburgh Handedness Inventory (Oldfield, 1971). They reported normal or corrected-to-normal vision and performed normally in a color vision test (Ishihara, 1917). Participants gave written informed consent before the experiment. All experimental procedures were in accord with the Declaration of Helsinki.

#### Stimuli and apparatus

A 22-in. computer screen with a refresh rate of 100 Hz was used for stimulus presentation. The screen was placed at a distance of 70 cm from the observer and its height was individually adjusted such that fixation was exactly at eye level for each participant. A button box was put centrally in front of the participants, with the left and right buttons of the box under their respective index fingers. Behind the box, a pedestal with a height of 14 cm was positioned and a wooden, custom-made cup was placed on a marked position at the middle of it, with its handle facing to the right. Stimulus delivery and experimental timing was controlled with Presentation Software (Neurobehavioral Systems, Albany, CA, USA).

The background for all stimuli was a colored virtual three-dimensional room with a table in it (**Figure 1**) and extended across the entire screen. Vanishing point perspective was used to create an impression of depth. A black dot surrounded by a black circle was placed on the table as fixation spot. Two different objects could appear on the table during the experiment: a cup and a cactus. The cup consisted of a photograph of the wooden cup that was on the pedestal during the experiment. The cactus matched size and shape of the cup and was adjusted for its mean luminance value. The objects were 7.5 cm wide and 7.2 cm high when presented on screen. Two objects were always shown simultaneously on the table, one virtually near, the other virtually far from the observer. The near and far objects started at a distance of 2.8° below and above the center of the fixation spot, respectively. They subtended a horizontal viewing angle of 2.5° to the left and 4.1° to the right. Note that the stronger extension toward the right was caused by the handle orientation to that side. Due to the identical physical size of the near and the far object, the latter was subjectively larger than the former.

There were four different display types (**Figure 1B**). Two of them contained identical objects (either two cactuses or two cups); the other two displays contained two different objects (one cup and one cactus). The go stimulus for grasp trials consisted of a single cup placed on the virtual table, subtending the same horizontal angles as in the two-objects presentation, but vertically placed exactly halfway in between the near and far positions. Stimulus creation and manipulation was realized using Gimp Version 2.8.

Grasping movements of the participants were recorded with a Polhemus Liberty electromagnetic motion tracker at a sampling rate of 240 Hz providing X, Y, and Z coordinates of each sensor in space. Motion sensors were attached to the right wrist and to the thumb and index finger of the right hand using adhesive tape. Motion data recording was controlled with custom Matlab scripts (Mathworks, Natick, MA, USA) and interfaced with Presentation software using the Matlab Workspace Extension implemented in Presentation.

#### Procedure

Each probe detection trial started with a fixation display for 1000 ms, during which the background stimulus, that is, the table in the virtual room, was visible to avoid abrupt visual changes upon the subsequent appearance of stimuli. Two objects then appeared on the table for 200 ms. Then, a probe (luminance change of the whole object) appeared on one of them for 100 ms, and subsequently the object returned to its original luminance. The other object did not change. Participants were instructed to press the corresponding button as quickly as possible in order to indicate the location of the luminance probe. Upon the participant’s reaction or after a maximum of 2000 ms in case no response was registered, an empty gray screen was presented for a random duration between 1000 and 1500 ms (inter-trial interval) before the next trial started. Assignment of left and right buttons to the object positions was counterbalanced among participants. Thus, there were eight different experimental conditions in the probe detection task, as shown from left to right in **Figure 1B**: cactus/cactus, cup/cup, cactus/cup, and cup/cactus, each of these in a probe-near and a probe-far version.

Grasp trials also started with 1000 ms fixation depicting the table. Upon the presentation of the go stimulus participants were to move their right hand toward the actual cup, grasp it by its handle, lift it in order to match the height of the cup on screen, and then put it back on the pedestal. The experimenter observed the movement and pressed a button as soon as the participants had returned with their right index finger to the right key of the button box. This initiated the inter-trial interval as described above, and then the next trial began.

The experiment consisted of 660 trials in total, among which 440 were probe detection trials and 220 were grasp trials. Trials were divided into 11 blocks of 60 trials each (40 probe detection, 20 grasp). After each block, participants received feedback about their mean reaction times and number of errors in the probe detection task during the last block. Each of the four stimulus displays appeared 110 times during the experiment, with half of the probes presented at the near and far positions, respectively. Stimulus sequence was randomized throughout the experiment and differed for each participant.

#### Data analysis

***Probe detection data.*** Trials with false or missing responses were excluded. Reaction times were computed from the onset of the probe until the button press was registered. For the purpose of outlier correction the experiment was divided into three parts to account for potentially slower reaction times in the first blocks or fatigue at the end of the experiment. The parts comprised the following blocks: part1: blocks 1–3, part 2: blocks 4–7, and part 3: blocks 8–11. Mean reaction times were calculated for each participant, condition, and part. Trials with reaction times exceeding ± 2 standard deviations from these individual means were excluded from the analysis (5% of trials on average).

After outlier correction, mean reaction times were computed separately for each subject in each of the eight conditions. Three factors were of interest in the current experiment: (1) *probe location*, that is, whether the probe appeared at the near or at the far position, (2) *object type*, that is, whether the probe appeared on the cup or on the cactus, and (3) *identity*, that is, whether two identical or two different objects (cup and cactus) were presented in a current trial. The inclusion of latter factor allowed us to elucidate whether the context in which an object is presented also affects probe detection. A 2 × 2 × 2 repeated-measures analysis of variance (ANOVA) with these within-subjects factors (*identity* – identical vs. different, *object type* – cup vs. cactus, and *probe location* – near vs. far) was computed on the reaction times. Error rates were very low in the present experiment (*M* = 2.36%, *SD* = 1.59) and were therefore not analyzed.

***Motion tracking data.*** Due to technical problems, in the case of three participants no motion data could be acquired. Therefore, only 32 participants were used for this analysis. Motion tracking data were processed with custom Matlab scripts. Movement onset was calculated separately for each trial as latency from the presentation onset of the go stimulus (a single, centrally presented cup) to the moment when the velocity of the wrist sensor exceeded 10 m/s and the index finger was at least 1 mm away from its starting position. Duration of the hand movement toward the cup was measured as the duration between movement onset and the point in time at which wrist velocity dropped below 10 m/s and the index finger was at least 20 cm away from its individual starting position. These criteria had to be fulfilled for a minimum of 10 consecutive sample points. Trials in which movement onset time was below 100 or above 1500 ms were excluded from further analysis. Movement durations below 200 ms or above 2000 ms also led to the exclusion of affected trials.

In order to compute the motion trajectories, a fourth-order low pass Butterworth filter with a cutoff frequency of 40 Hz was applied to the data. The motion tracking data between movement onset and the end of the movement were subsequently time-normalized in terms of percentages, 0% reflecting movement onset and 100% reflecting the end of the movement, that is, the touch of the object.

### RESULTS

#### Probe detection data

**FIGURE 2:**
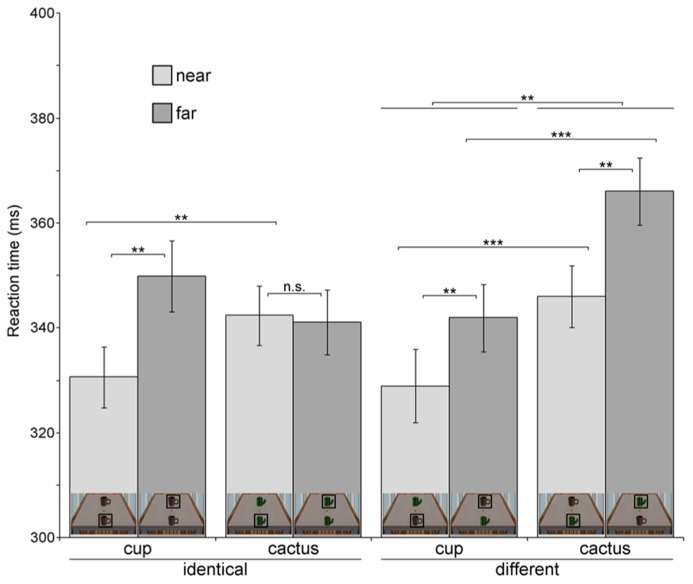
**Reaction times in the probe detection task for each of the eight experimental conditions (Experiment 1).** For clarity, miniature pictures of the stimulus corresponding to each condition are shown at the bottom of each bar. The object at which the luminance probe appeared in a specific condition is marked with a black square. Error bars denote ± 1 SEM. Significance levels are indicated for effects of interest. **p < 0.01, ***p < 0.001, n.s.-not significant.

**Table 1** and **Figure 2** provide an overview of all eight conditions. Probes which appeared in near space (*M* = 337.00 ms, *SD* = 36.53) were responded to faster than probes in far space (*M* = 349.75 ms, *SD* = 38.98), reflected in a significant main effect of probe location, *F*(1,34) = 7.236, *p* = 0.011, η^2^ = 0.175. When the probe appeared on the cup (*M* = 337.86 ms, *SD* = 38.95), participants detected it more quickly than when it appeared on the cactus (*M* = 348.88 ms, *SD* = 36.84), as indicated by the significant main effect of object type, *F*(1,34) = 11.114, *p* = 0.002, η^2^ = 0.246. A significant two–way interaction emerged between the factors identity and object type, *F*(1,34) = 10.929, *p* = 0.002, η^2^ = 0.243. To follow-up on this result, we computed separate ANOVAs for displays with identical objects and displays containing different objects. In the identical-objects condition, probes appearing on the cup (*M* = 340.26 ms, *SD* = 33.04) were not detected significantly faster than at the cactus (*M* = 341.73 ms, *SD* = 30.31; *F* < 1, *p* > 0.47). In the different objects condition, that is, when one cup and one cactus were presented simultaneously, participants detected probes appearing on the cup (*M* = 335.47 ms, *SD* = 36.23) faster than on the cactus (*M* = 356.04 ms, *SD* = 32.65), independent of their location, *F*(1,34) = 12.309, *p* = 0.001, η^2^ = 0.266. This reaction time increase for probes appearing at the cactus in the different objects setting was also visible in the main effect of identity, *F*(1,34) = 11.990, *p* = 0.001, η^2^ = 0.261 (identical: *M* = 341.00 ms, *SD* = 36.45, different: *M* = 345.75 ms, *SD* = 39.95).

**Table 1:** Mean reaction times in all eight conditions, Experiment 1.

Condition	Identical				Different			
	Cup		Cactus		Cup		Cactus	
	Probe near	Probe far	Probe near	Probe far	Probe near	Probe far	Probe near	Probe far
Mean RT	330.62	349.90	342.38	341.08	328.96	341.97	346.02	366.05
(SD) in ms	(34.29)	(39.78)	(33.69)	(36.65)	(41.33)	(37.87)	(34.87)	(37.92)

*Note: the order of conditions in this table is the same as in **Figure 2**.*

Most importantly, the three-way interaction between identity, object type, and probe location was significant, *F*(1,34) = 23.560, *p* < 0.001, η^2^ = 0.409. To test our hypothesis of a reaction time advantage for probe detection at the cup as compared to the cactus in near space, we computed an identity × object type ANOVA for near space probes. As hypothesized, probes appearing on the cup in near space were detected faster than probes appearing on the cactus at the same spatial position (see **Table 1**). This was reflected in a significant main effect of object type, *F*(1,34) = 16.115, *p* < 0.001, η^2^ = 0.322. The identity × object type interaction was not significant in near space (*F* < 1, *p* > 0.37), indicating that probe detection was generally faster at the near cup compared to the near cactus, independent of whether another cup or a cactus was simultaneously present in far space. To test the second part of our hypothesis, namely whether probes at the near cup were detected more quickly than at the far cup, we computed an additional one-factorial ANOVA in which probe detection at the near cup was statistically compared to probe detection at the far cup, independent of identity. As predicted, probe detection at cups in near space (*M* = 329.79 ms, *SD* = 36.62) was faster than in far space (*M* = 345.94 ms, *SD* = 35.83), *F*(1,34) = 10.746, *p* = 0.002, η^2^ = 0.240.

In far space, the identity × object ANOVA yielded a significant interaction between both factors, *F*(1,34) = 4.673, *p* = 0.038, η^2^ = 0.12. In the identical-objects condition, probes appearing at the far cactus were reacted to faster than probes appearing at the far cup (see **Table 1**), *F*(1,34) = 4.673, *p* = 0.038, η^2^ = 0.121. This pattern was reversed in the different objects condition, with faster probe detection at the far cup than the far cactus, *F*(1,34) = 13.813, *p *= 0.001, η^2^ = 0.289. Thus, there was no general probe detection advantage for cups in far space. The results furthermore indicate that responses were significantly slowed for probes appearing on the cactus at the far position when a cup was simultaneously present in near space.

To sum up, our results confirm a reaction time advantage for probe detection at the near cup, both compared to probe detection at the cactus in near space and at the cup in far space. Additionally, in the presence of the near cup probe detection times at the far cactus were significantly increased.

To explore whether button assignment influenced reaction times in terms of a facilitation of right-hand responses to near graspable stimuli (Costantini et al., 2011a), we conducted an additional ANOVA, which included the twofold between-subjects factor “button assignment.” This analysis revealed only a marginally significant probe location × button assignment interaction, *F*(1,33) = 3.831, *p* = 0.059, η^2^ = 0.104. Therefore, faster right-hand responses to the near cup could not be statistically confirmed.

#### Motion tracking data

On average, < 1% (*SD* = 0.88) of grasp trials were discarded due to premature or very slow initiation of the movement. Furthermore, 1.68% (*SD* = 4.43) of grasp trials were excluded after applying the minimum and maximum movement duration criterion. Grasping movements were initiated on average 695.33 ms (*SD* = 79.44) after the onset of the go stimulus and had a mean duration of 752.17 ms (*SD* = 195.59). Average movement onset latency and duration were correlated with the reaction times in each of the eight probe detection conditions using Pearson correlations. This analysis did not yield any significant results (uncorrected *p*s > 0.06).

**FIGURE 3:**
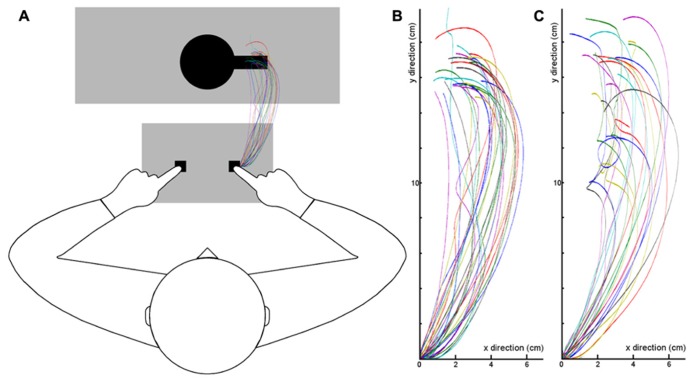
**Mean motion trajectories of the index finger for each participant (single lines depict single participants).** **(A)** Trajectories in Experiment 1, plotted into the experimental setup. Participants started their movements from the right key of the button box and moved toward the cup which was on the pedestal (big gray shade). **(B)** The diagram shows the same finger trajectories in a two-dimensional coordinate system (X- and Y-coordinates), 0 represents the starting point of the grasping movement. **(C)** Finger trajectories in Experiment 2.

**Figure 3** shows the averaged motion trajectories of the index finger for each participant. As can be seen, the motion tracking data confirm that participants performed the grasping task in an appropriate manner.

### DISCUSSION

In the present experiment we aimed to investigate how object affordance influences the allocation of visual attention as a function of object distance. In line with our predictions, probes appearing at an affording object (a cup) were reacted to faster in near space than in far space. Furthermore, this reaction time advantage at reachable distance was only observed for probes on the cup, but not for probes on a physically matched non-affording object (a cactus).

We interpret this reaction time advantage as indirect evidence that attention was preferentially allocated to the near cup, tracing back to the idea that a probe is detected faster when it appears at a currently attended location (Posner, 1980; Humphreys et al., 2004). The point in time at which we presented the probe (200 ms after stimulus onset) is thought to tap initial allocation of selective visual attention, which can be measured in the N2pc component of the ERP (Luck and Hillyard, 1994; Eimer, 1996). We therefore consider the reaction time advantage to probes at the near cup an indirect reflection of initial attention deployment to the near cup. Thus, our data are in line with the idea that graspable objects preferentially attract attention when they appear at a spatial location important for grasping (Handy et al., 2003). Moreover, our results fit previous reports in the literature showing a near-space preference for graspable objects (Gallivan et al., 2009, 2011; Cardellicchio et al., 2011; Costantini et al., 2011a,b).

## EXPERIMENT 2

The data from Experiment 1 are in line with the idea that an affording object in near space might result in an attention bias toward that object; however the results cannot tell apart whether the near-space preference for the cup was due to its affordance *per se* (as opposed to the clearly non-affording cactus) or due to its arrangement in a graspable position, with a right-facing handle.

Behavioral studies indicate that the way in which handled objects are arranged visually influences motor reactions. Right-hand responses are executed faster when an object is presented with its handle facing to the right and vice versa, even when handle orientation is completely task-irrelevant (Tucker and Ellis, 1998; Costantini et al., 2010, 2011a; Goslin et al., 2012). This pattern is interpreted in terms of an activation of the hand which would be used for actually grasping the object, and it has been referred to as affordance effect (Riggio et al., 2008) or spatial alignment effect (Costantini et al., 2010, 2011a) in the literature. Even though these effects are not always easy to disentangle from spatial compatibility effects (Simon and Rudell, 1967), studies have indicated that spatial compatibility effects and affordance-related reaction time effects may be at least partly dissociable (Pellicano et al., 2010; Cho and Proctor, 2013). In contrast to the evidence on handle-hand correspondence, other studies suggest that right-handers prefer objects ready to the right hand, such that they are immediately graspable with their preferred hand (Buccino et al., 2009; Gallivan et al., 2009, 2011). Importantly, the visuomotor response to objects that are immediately graspable with the right hand is enhanced compared to objects graspable with the left hand (Gallivan et al., 2009, 2011). Thus, if the visuomotor response leads to an attention bias as proposed by Handy et al. (2003), a manipulation of handle orientation in near space would influence probe detection performance.

To shed more light on this issue, Experiment 2 included objects with handles to the left. A cup with a left-facing handle at the near position would not appear immediately graspable to a right-hander. We therefore hypothesized that in the case of left-facing handles, probes appearing on the near cup would not be detected faster than probes on the far cup or on the near cactus.

### MATERIALS AND METHODS

#### Participants

Twenty-six students from the Philipps University Marburg participated in the second experiment for course credit. Data sets from three participants were discarded, two of them due to technical problems during the measurement. Another participant reported problems with three-dimensional vision and was therefore also excluded. The remaining 23 participants (19 female) had a mean age of 22.35 years (*SD* = 3.59) and were right-handed according to the Edinburgh Handedness Inventory (Oldfield, 1971). They reported normal or corrected-to-normal vision and performed normally in a color vision test (Ishihara, 1917). Participants gave written informed consent before the experiment. All experimental procedures were in accord with the Declaration of Helsinki.

#### Stimuli and apparatus

The experimental setup was identical to Experiment 1. The same held true for the stimuli; however the four stimulus displays for the probe detection task used in Experiment 1 (see **Figure 1B**) were now also present with the handles of both objects oriented to the left, subtending a horizontal viewing angle of 2.5° to the right and 4.1° to the left.

#### Procedure

The inter-trial interval was shortened to a random duration between 250 and 750 ms in order to include more trials. Because of the additional variation of handle orientation in probe detection trials, we reduced the number of identical-objects trials to a total of 176, equally distributed across cactus and cup and the two handle orientations. Each of the four different-objects displays (cup near-cactus far, cactus near-cup far in both orientations) was presented 110 times throughout the experiment. The experiment thus consisted of 836 trials in total, of which 616 were probe detection trials and 220 were grasp trials. Trials were divided into 11 blocks of 76 trials each (56 probe detection, 20 grasp). After each block, participants received feedback about their mean reaction times and number of errors in the probe detection task during the last block.

Apart from these changes, the procedure was identical to Experiment 1.

#### Data analysis

Probe detection data were analyzed as in Experiment 1. The exclusion of reaction time outliers in the probe detection task affected 5.1% of trials on average (*SD* = 1.83). For data analysis, three factors were of interest in the current experiment: (1) *probe location*, that is, whether the probe appeared at the near or at the far position, (2) *object type*, that is, whether the probe appeared on the cup or on the cactus, and (3) *orientation*, that is, whether the handles were oriented to the left or right. A 2 × 2 × 2 repeated-measures ANOVA with these within-subjects factors was computed on reaction times. Only trials with different-stimulus displays were used for analysis in the present experiment, due to the low number of identical-stimulus displays. Error rates were again very low (*M* = 3.4%, *SD* = 2.12) and were therefore not analyzed.

Motion data analysis was identical to Experiment 1. The motion tracker failed to record data in the case of one participant, and another participant exceeded the maximum movement duration in 63% of the trials and was therefore excluded from this analysis. Thus, the movement data were calculated on a set of 21 participants.

### RESULTS

#### Probe detection data

**FIGURE 4:**
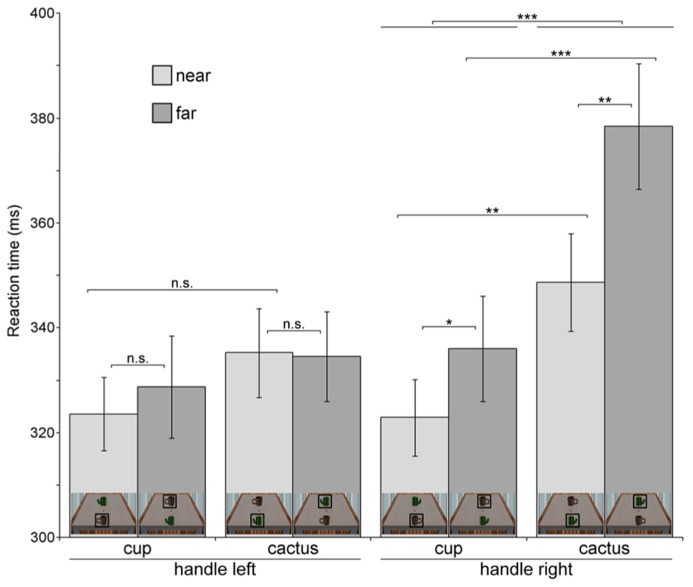
**Reaction times in the probe detection task for each of the eight experimental conditions (Experiment 2).** For clarity, miniature pictures of the stimulus in each condition are shown at the bottom of each bar. The object at which the luminance probe occurred in a specific condition is marked with a black square. Error bars denote ± 1 SEM. Significance levels are indicated for effects of interest. *p < 0.05, **p < 0.01, ***p < 0.001, n.s.-not significant.

**Table 2** and **Figure 4** provide an overview of all eight conditions in Experiment 2. First and foremost, the three-way interaction (object type × probe location × orientation) was significant, *F*(1,22) = 6.737, *p* = 0.017, η^2^ = 0.234. We therefore tested the object type × probe location interaction separately for the two handle orientations.

**Table 2:** Mean reaction times in all eight conditions, Experiment 2.

Condition	Handle left				Handle right			
	Cup		Cactus		Cup		Cactus	
	Probe near	Probe far	Probe near	Probe far	Probe near	Probe far	Probe near	Probe far
mean RT	323.54	328.74	335.24	334.57	322.90	336.04	348.72	378.42
(SD) in ms	(33.53)	(46.94)	(40.75)	(41.31)	(35.01)	(48.30)	(44.48)	(57.43)

*Note: the order of conditions in this table is the same as in **Figure 4**.*

For handles oriented to the right, the pattern reported in Experiment 1 could be replicated, with a significant object type × probe location interaction, *F*(1,22) = 4.339, *p *= 0.049, η^2^ = 0.165. Probes which appeared on the near cup were detected faster than on the near cactus, *t*(22) = 3.572, *p* = 0.001, and faster than on the far cup, *t*(22) = 1.942, *p* = 0.033. In addition, reactions to probes on the far cactus were significantly slower than reactions to probes on the far cup, *t*(22) = 5.99, *p* < 0.001, which replicates the reaction time increase also observed in Experiment 1. For handles oriented to the left, the object type × probe location interaction was not significant (*p* = 0.35).

The three-way ANOVA also revealed a significant probe location × orientation interaction (*F*(1,22) = 13.222, *p* = 0.001, η^2^ = 0.375). Near probes were generally detected faster than far probes when handles were oriented to the right (near: *M *= 335.81 ms*, SD *= 36.08; far: *M* = 357.23 ms, *SD* = 50.27), *F*(1,22) = 7.807, *p* = 0.011, η^2^ = 0.262; for left-oriented probes there was no significant main effect of target location (*p* = 0.655). Thus, the data for right-oriented objects are in accord with Experiment 1.

Additionally, analysis of the object type × orientation interaction, *F*(1,22) = 78.736, *p* < 0.001, η^2^ = 0.782, revealed that for objects oriented to the right, probes appearing on the cup were responded to faster than on the cactus (cup: *M* = 329.47 ms, *SD* = 38.93; cactus: *M* = 363.57 ms, *SD* = 45.21), *F*(1,22) = 32.898, *p* < 0.001, η^2^ = 0.599, in line with results from Experiment 1. Descriptively, this also held true for left-oriented handles, but the effect was not significant (*p* = 0.169).

The faster reaction times for probes appearing on the cup as compared to the cactus were also evident in a significant main effect of object type in the three-way ANOVA, *F*(1,22) = 13.269, *p* = 0.001, η^2^ = 0.376. Furthermore, responses were generally faster in trials with left-oriented (*M* = 330.52 ms, *SD* = 35.46) as compared to right-oriented handles (*M* = 346.52 ms, *SD* = 39.71). This pattern was reflected in a significant main effect of orientation, *F*(1,22) = 63.122, *p* < 0.001, η^2^ = 0.742.

We also explored whether button assignment influenced reaction times, in a way that participants who pressed the right button for near stimuli would be faster with cup handles to the right, as compared to participants with the left button assigned to near probes, who might experience reaction time facilitation by a left-facing cup handle (Costantini et al., 2011a). We conducted an additional ANOVA, which included the twofold between-subjects factor “button assignment.” However, the object type × probe location × orientation × button assignment interaction was non-significant (*p* > 0.66).

#### Motion tracking data

Outlier correction of the movement data led to the exclusion of 1.37% (*SD* = 2.42) of trials on average due to reaction time errors; 2.86% (*SD* = 4.02) of the grasp trials were discarded because they did not meet the inclusion criteria for the minimally and/or maximally admitted movement duration. Grasping movements were initiated on average 742.42 ms (*SD* = 94.18) after the onset of the go stimulus and had a mean duration of 841.70 ms (*SD* = 229.72). No significant correlations between these two motor variables and reaction times in the eight probe detection conditions were observed (uncorrected *p*s > 0.07).

The motion tracking data again confirm that participants performed the grasping task appropriately. The mean trajectories of each participant can be seen in **Figure 3C**.

## GENERAL DISCUSSION

Based on different studies indicating a stronger visuomotor response to affording objects in near as compared to far space (Gallivan et al., 2009, 2011; Costantini et al., 2010, 2011a,b; Cardellicchio et al., 2011), the present set of experiments used a probe detection task to investigate whether initial deployment of visual attention is stronger to graspable than non-graspable objects in near space, and whether such difference also holds true for a graspable object in near as compared to the same object in far space. Such pattern was in fact revealed in Experiment 1: probe detection was fastest when probes appeared at the cup in near space, which indicated that attention was preferentially allocated to the near, affording object. In the second experiment we could show that this reaction time advantage for probe detection at the near cup was no longer present with handles facing to the left.

As suggested by Handy et al. (2003), graspable objects which appear at locations important for grasping may draw attention even when they are task-irrelevant. This could happen because observers implicitly recognize an object’s potential for action, thereby leading to an attentional bias toward that object. With the present set of experiments, we were able to extend previous findings by showing that such attention bias can also be observed as a function of object distance.

From our results, we can conclude that the attention bias induced by the near, graspable cup cannot be explained by attentional capture due to basic physical stimulus differences between our two object types. In fact, cup and cactus were matched for size, shape, luminance, and orientation, all of which are attributes that undoubtedly or very likely capture attention in a bottom-up fashion (Wolfe and Horowitz, 2004). The only basic attribute which might still work in such an attention-capturing fashion would be the object’s color. However, two observations in our study allow us to rule out this possibility: on the one hand, if one color captured attention more than the other, this would also be evident in reaction times to far space probes; however, the results pattern was rather mixed in Experiment 1: neither the probes appearing on the cup nor those appearing on the cactus had a clear advantage in far space. Furthermore, in Experiment 2 there was no significant reaction time difference between probes on the cactus and probes on the cup even in near space, when handles were oriented to the left. This would provide additional evidence against the idea of bottom-up attentional capture by mere physical stimulus differences.

Instead, the pattern we reported in Experiment 2 clearly points to the role of immediate graspability of an affording object, such that only when the object is “ready to hand” an attentional bias toward it is induced (Handy et al., 2003; Handy and Tipper, 2007). This is in line with a study by Buccino et al. (2009), who reported that MEPs at the right hand were significantly higher for objects with an intact handle as compared to a broken one, but only when objects were oriented to the right. Thus, not only affordance *per se* (in terms of the affording cup compared to the non-affording cactus in our experiment) is crucial for recognizing action potentials, but also the possibility to immediately interact with an object. This possibility, in turn, appears to be influenced by object distance as well as handle orientation. Here, it is particularly interesting to consider the case of a patient with lesions in parietal cortex, who had problems recognizing the action affordance of an object when its handle was oriented away from him, but whose performance benefitted significantly when the handle orientation was adjusted such that the object appeared immediately graspable to him (Humphreys and Riddoch, 2001). Thus, in the light of these findings the present results are in accord with the idea that not only object characteristics *per se*, but rather their potential for immediate action may bias attention toward an affording object. However, even though the object type × probe location interaction was clearly non-significant for objects with left-oriented handles in Experiment 2, from a merely descriptive point of view, participants were fastest at responding to probes on the near cup in the handle-left condition as well (see **Figure 4**). Therefore, it is possible that the near cup, even though not immediately graspable, still may retain some of its behavioral relevance because it is close to the observer.

In both experiments, participants responded exceptionally slow to probes at the cactus in far space while a cup was simultaneously present at the near position. We had initially not predicted such effect; however, it would also be in line with an attentional account of the present data. The reaction time increase in latter condition can be interpreted in terms of a strong attention bias toward the near cup, and subsequently increased costs of shifting the focus to the far cactus. Such strong reaction time increase was not observed when participants had to react to the far cup while there was also a cup at the near position (Experiment 1). It appears that engagement of attention (Posner and Petersen, 1990) by the near cup was comparable in identical-objects and different-objects contexts, reflected in almost identical mean reaction times in both conditions. However, when the probe was presented at the far location, the reaction time difference between near and far was 19 ms in the identical-objects condition containing two cups, contrasted with 37 ms in the different-objects condition with the cup at the near position and the cactus at the far one. According to Desimone and Duncan (1995), objects in our environment compete for selective visual attention, a process which may be biased, among others, by their behavioral relevance. In this vein, the far cup would still have some behavioral relevance, but the cactus would not. This, in turn, would increase the competition between cup and cactus specifically when the cup appears immediately graspable. In line with this interpretation, in the display with two cactuses probe detection latencies were highly comparable for near and far, suggesting that no attention bias was present. The same is true for probe detection at the far cactus with handles oriented to the left. Due to the apparent lack of immediate behavioral relevance of the left-handled cup in near space, no attention bias was induced toward it, and therefore no reaction time increase could be observed.

The selective reaction time advantage for probes at right-oriented cups in near space allows us to rule out a general lower visual field preference as explanation for our results. In the present set of experiments, near space was always located below fixation while far space was located above. The reduction of reaction times to the cup in near space is therefore also in line with research supporting a lower visual field preference for grasping (Rossit et al., 2013), and it makes perfect sense that the cup advantage disappeared when it was oriented to the left and therefore not immediately graspable. Thus, our results support enhanced processing of immediately graspable objects at a location important for grasping, namely the lower visual field (Handy et al., 2003). However, even though the factors of distance on the one hand and upper/lower visual field on the other cannot be disentangled in the present experiment, they seem to be partly independent of each other. For example, enhanced activation in the SPOC during passive viewing of graspable objects in near space compared to far is also observed with all objects located below fixation (Gallivan et al., 2009, 2011). Research using methods with a higher temporal resolution than fMRI are needed to gain more insight into the mechanisms triggered by object distance and graspability on the one hand, and upper versus lower visual field on the other.

One might argue that the probe detection advantage at the cup could be due to a more frequent appearance of the cup on screen as compared to the cactus, because it was also presented on grasp trials. We acknowledge that the more frequent presentation of the cup during the experiment may cause higher familiarity with the cup than the cactus. It is also reasonable to assume that cups are generally more familiar to participants than cactuses due to everyday experience. However, we do not consider familiarity a likely explanation for our results, because there was no overall reaction time advantage for the cup, which would be expected from the familiarity interpretation. In Experiment 1, faster reactions to the cup were corroborated in near space, but in far space the pattern was not that clear. In Experiment 2, no significant reaction time difference between these two objects emerged when handles were oriented to the left. Furthermore, research suggests that high familiarity or motor experience with an object may in fact reduce the visuomotor response to it (Handy et al., 2006).

The failure to find a significant interaction with button assignment in our data seems to be at odds with findings from Costantini et al. (2010, 2011a). These authors reported that right-hand responses to a cup with its handle facing to the right were executed faster than left-hand responses, but only in near space. With handles facing left, this pattern was reversed. Thus, in the present experiment those participants who pressed the right button for probes appearing on right-oriented cups in near space should have had a reaction time advantage in this condition, compared to participants who pressed the left button for near space probes, who would be faster with cup handles facing to the left. Our data did not support such pattern, suggesting that a near object with a right-facing handle does not necessarily facilitate right-hand responses and vice versa. On the one hand, this may depend on the action which is executed. In the present study, participants pressed a button while Costantini et al. (2011a) had their participants perform pantomime movements. Furthermore, we varied button assignment as a function of distance, but not handle orientation in the present experiment. Therefore it is not possible to directly compare reactions to different handle orientations within-subjects considering only near-space objects. Moreover, while several studies report that handle orientation facilitates responses with the corresponding hand, including button presses (Tucker and Ellis, 1998; Buccino et al., 2009; Goslin et al., 2012), the TMS study by Cardellicchio et al. (2011) showed generally enhanced MEPs at the right hand for near, graspable stimuli independent of handle orientation. To sum up, the evidence on handle-hand correspondence is somewhat equivocal; however, this also shows that object affordance might in fact be much more than just spatial compatibility.

In Experiment 2 we observed a main effect of handle orientation, which was characterized by generally faster probe detection when handles were oriented toward the left as compared to the right. One possible explanation for this observation is that due to the lack of action relevance of objects with handles oriented to the left, no attention bias toward the cup could be induced and thus the object competition model adapted in our experiments (Handy et al., 2003) would not result in competition between near and far objects (Desimone and Duncan, 1995). Another reason might be interference between the go cue for grasping (a single, central cup with its handle oriented to the right) and probe detection on right-oriented objects. In fact, one participant reported after the experiment that she had experienced the condition with handles toward the left as easier than responding to right-oriented objects because of their similarity with the go stimulus. Even though many other participants reported afterward that they were not aware of the variation of handle direction, such subtle response interference might still be present in the data.

In sum, our results fit the literature showing a near-space advantage for graspable over non-graspable objects, both when comparing an affording object to a clearly non-affording one, and also when the immediate graspability of an affording object is manipulated. Specifically, we could show that this near-space advantage for graspable objects goes along with an attention bias toward that object. Therefore, the data are in line with the idea of differential attention allocation to objects depending on their potential for action (Handy et al., 2003). However, in order to test this attentional account of the present data more directly, ERP studies are needed to gain more insight into the processes triggered by the objects and their respective positions in space.

## Conflict of Interest Statement

The authors declare that the research was conducted in the absence of any commercial or financial relationships that could be construed as a potential conflict of interest.
